# Genetic predisposition to nonalcoholic fatty liver disease: insights from ANGPTL8 gene variants in Iranian adults

**DOI:** 10.1186/s12944-023-01905-0

**Published:** 2023-09-07

**Authors:** Samira Saghafi, Elham Chamani, Fatemeh Salmani, Reza Fadaei, Efat Shafiei, Nariman Moradi, Tahmine Tavakoli

**Affiliations:** 1 Student Research Committee, Birjand University of Medical Sciences, Birjand, Iran; 2 Department of Clinical Biochemistry, Faculty of Medicine, Birjand University of Medical Sciences, Birjand, Iran; 3 Department of Epidemiology and Biostatistics, Social Determinants of Health Research Center, Faculty of Health, Birjand University of Medical Sciences, Birjand, Iran; 4 Sleep Disorders Research Center, Kermanshah University of Medical Sciences, Kermanshah, Iran; 5 Student Research Committee, Kurdistan University of Medical Sciences, Sanandaj, Iran; 6 Cellular and Molecular Research Center, Research Institute for Health Development, Kurdistan University of Medical Sciences, Sanandaj, Iran; 7 Cardiovascular Research Center, Birjand University of Medical Sciences, Birjand, Iran

**Keywords:** NAFLD, ANGPTL8, Polymorphism, Lipid profile

## Abstract

Nonalcoholic fatty liver disease (NAFLD) is a prevalent chronic liver disease with a global prevalence, and modulation of ANGPTL8 expression has emerged as a promising predictor of NAFLD susceptibility. This research was conducted to scrutinize ANGPTL8 protein expression in NAFLD patients and elucidate the interplay between ANGPTL8 gene polymorphisms and their lipid profiles, thus shedding new light on the pathophysiology of this complex disease. The study comprised 423 unrelated participants, including 222 healthy controls and 201 individuals with NAFLD, screened using FibroScan/ultrasonography and laboratory tests. The main goal focused on the genotype and allele frequency distribution in the ANGPTL8 gene, specifically analyzing two genetic variations: rs737337 (T/C) and rs2278426 (C/T). The participants diagnosed with NAFLD were slightly younger (*P* ≥ 0.05) and had a higher body mass index (BMI) than the individuals in the control group. Notably, there was a significant difference in the occurrence of the rs737337 polymorphism between the NAFLD and control groups, with a lower frequency observed in the NAFLD group. Our results indicated that individuals with the TC + CC genotype and C allele of rs737337 (T/C) had a decreased risk of higher levels of ALT and AST. Conversely, those with the CT, CT + TT genotype, and T allele of rs2278426 (C/T) exhibited an increased risk of higher levels of ALT and AST. The results imply that the rs2278426 (C/T) variant of the ANGPTL8 gene is more strongly linked to an increased risk of NAFLD compared to the rs737337 polymorphism. However, additional research is needed to understand the specific molecular mechanisms responsible for the upregulation of ANGPTL8 in individuals with NAFLD.

**Supplementary Information:**

The online version contains supplementary material available at 10.1186/s12944-023-01905-0.

## Introduction

Nonalcoholic fatty liver disease (NAFLD) stands out as the predominant etiology of liver-related disorders and has emerged as an imperative public health problem worldwide. Hence, wide research attention has been directed toward NAFLD and its more severe form, nonalcoholic steatohepatitis (NASH). The number of NAFLD cases in the United States is expected to rise from 83.1 million in 2015 (25% of the population) to 100.9 million by 2030. NASH will account for a more significant proportion of these instances, rising from 20 to 27% of people with NAFLD throughout this time period [1]. NAFLD is typically regarded as the hepatic manifestation of metabolic syndrome and is linked with metabolic comorbidities such as obesity, type 2 diabetes mellitus (T2DM), or dyslipidemia in the majority of individuals [2]. Although NAFLD is prevalent and has significant consequences, its monitoring and diagnosis primarily depend on expensive imaging methods such as ultrasonography or blood tests that measure liver enzyme profiles, often leading to inaccurate results. This difficulty emphasizes the urgent requirement for precise biomarkers to identify NAFLD [3].

Numerous studies have been conducted to unravel the molecular underpinnings of NAFLD, revealing significant insights [4]. In this regard, the identification of a TM6SF2 variant linked to NAFLD susceptibility, its crucial involvement in very low-density lipoprotein (VLDL) lipidation, and its association as a risk factor for cardiovascular disease provide compelling evidence for the key role of TM6SF2 in the pathogenesis of NAFLD [5, 6].

Angiopoietin-like protein 8 (ANGPTL8), also referred to as lipasin, RIFL, C19orf80, TD26, or betatrophin, is a hepatokine that is naturally regulated. It decreases expression levels during fasting and increases when the body is well fed. Recent studies have demonstrated that this protein plays a crucial role in lipid metabolism. When the ANGPTL8 gene is overexpressed using adenoviral vectors in mice, it leads to elevated levels of triacylglycerol (TG) in the bloodstream. On the other hand, mice that lack the ANGPTL8 gene, known as ANGPTL8-knockout animals, have lower concentrations of TG in their plasma [7, 8]. ANGPTL8 inhibits LPL activity in humans by interacting with ANGPTL3 or ANGPTL4 [7, 9], and various ANGPTL8 gene variations have been associated with lipid changes [10–12]. Significantly, the levels of ANGPTL8 in the bloodstream are lower in various metabolic disorders, such as obesity, type 2 diabetes (T2D), and dyslipidemia. However, there are contrasting findings regarding ANGPTL8 concentrations following bariatric surgery, with some studies reporting higher levels. Conversely, higher levels of ANGPTL8 expression in the blood and liver are associated with various stages of NAFLD [13]. Although studies have found that blood ANGPTL8 levels are higher in NAFLD patients [14], the correlation between ANGPTL8 and the lipid profile in the etiopathogenesis of NAFLD remains unexplained.

ANGPTL8 has three sequence variants that have been connected to lipid metabolism [15]: the SNP rs2278426 denotes an SNP (CGG to TGG) where tryptophan (W) replaces the amino acid arginine (R) [16]. This SNP’s minor allele frequency (MAF), which fluctuates depending on ethnicity, represents 15% of the populations studied by the 1000 Genome Project, 26% of Hispanics, 18% of African Americans, and 5% of European Americans [16, 17]. A synonymous mutation in DOCK6 upstream of the ANGPTL8 transcription start site is attributed to the second single nucleotide polymorphism (SNP) known as rs737337 (T to C). Additionally, at position 12,118, the third SNP, identified as rs145464906 (CAG to TAG), results in an early stop codon [18]. Recent studies have demonstrated that this variation in ANGPTL8 in metabolic syndrome is associated with a lower risk of hypercholesterolemia, hyperglycemia, insulin resistance, etc. [11].

The serum level of ANGPTL8 has been connected to NAFLD in previous research [3]; however, there are still few known studies at the level of ANGPTL8 gene variations, particularly in populations such as those in developing countries where NAFLD incidence is high.

This study examined the impact of two primary variants of the ANGPTL8 gene on NAFLD phenotypes in adult Iranians. This research aimed to shed light on the role of these ANGPTL8 gene variants in NAFLD and their involvement in regulating lipid and glucose metabolism in the Iranian population.

## Material & method

### Study population

A total of 423 Iranian adults (222 healthy controls and 201 with NAFLD) were recruited for the study with the approval of the Ethics Committee of Birjand University of Medical Sciences (IR.BUMS.REC.1400.152).

Our study focused on adult participants aged 20 years or older. To ensure the accuracy of our findings, we excluded individuals who were classified as high alcohol consumers, which was defined as an average daily consumption of 20 g or more for women and 30 g or more for men. Additionally, we excluded participants who had a diagnosis of diabetes or an unknown diabetes status, autoimmune or viral hepatitis, infectious illnesses, renal disease, or cancer. Furthermore, individuals who were taking statins or other medications intended to lower fat or cholesterol levels were not included in the study.

### NAFLD diagnosis

Initially, we primarily utilized sonography and liver enzyme levels for the diagnosis of NAFLD. Furthermore, liver stiffness measurements (LSM) were performed by vibration-controlled transient elastography (VCTE) using FibroScan (Echosens, Paris, France). An experienced physician performed LSM on all patients after a minimum of 8 h of fasting and following liver ultrasonography. Additional information about the technical background and examination procedures can be found in a previous publication [19]. Clinically significant or advanced hepatic fibrosis was defined as an LSM value of ≥ 7 kPa (equivalent to Kleiner fibrosis stage ≥ F2 on histology) or an LSM value of ≥ 8.7 kPa (equivalent to ≥ F3 on histology) [20, 21].

### Anthropometric measurements, blood samples, and biochemical evaluations

The study utilized anthropometric data retrieved from the database, which included several measurements: height (in centimeters), weight (in kilograms), and blood pressure (in mmHg). We employed the following formula to evaluate BMI: weight in kilograms divided by the square of height in meters. Blood and serum samples were collected from patients and control participants after an overnight fast and properly stored for subsequent analysis. To measure fasting blood glucose (FBG), total cholesterol, high-density lipoprotein (HDL) cholesterol, low-density lipoprotein (LDL) cholesterol, remnant cholesterol (RC; which was calculated by subtracting LDL and HDL from total cholesterol), triglycerides (TG), insulin, and the homeostasis model assessment of insulin resistance (HOMA-IR), biochemical analysis was conducted using commercially available kits on an automated biochemical analyzer (Konelab, Espoo, Finland). For the measurement of serum concentrations of aspartate transaminase (AST) and alanine transaminase (ALT), kinetic diagnostic methods were employed using a kit from Pars Azmoon Inc. (Tehran, Iran). Genetic analysis was performed using whole blood samples. To determine serum levels of ANGPTL8, we employed a commercially available human ELISA kit (Eastbiopharm, China) with an intra-assay variance of 8% and an interassay variance of 8%. The ELISA procedure followed the manufacturer’s instructions for accurate and reliable measurements.

### Genetic analysis

The ARMS-PCR technique was employed to separate alleles at rs892066 (C/G). ARMS-PCR experiments were carried out in two tubes using a PCR kit (Yekta Tajhiz Azma, Iran) and the following primers: common primer 5′- ATTGTGCGGCCATAGAGACC − 3′, specific primers 5′- TGCCTGCTCTGCCTGATC − 3′, and 5′- TGCCTGCTCTGCCTGATG − 3′. The PCR cycling conditions for the ARMS-PCR experiments consisted of 30 cycles. Each cycle included denaturation at 95 °C for 30 s, annealing at 58 °C for 45 s, and extension at 72 °C for 40 s. After 30 cycles, a final extension step was conducted at 72 °C for 7 min. The PCR began with an initial denaturation step at 95 °C for 4 min. To confirm the results, the ARMS-PCR product, which had a length of 214 bp, was subjected to electrophoresis on a 2% agarose gel. In heterozygotes (CG), the ARMS-PCR product was visible in both microtubes. However, in homozygotes (CC or GG), the ARMS-PCR product was only visible in one microtube.

Subsequently, the ARMS-PCR products with a length of 214 bp were used for genotyping rs2278426 (C/T) using the Restriction Fragment Length Polymorphism (RFLP) technique. The ARMS-PCR products were subjected to digestion with the BtsCI restriction enzyme obtained from NEB (New England Biolabs). The digested products were then separated by electrophoresis on a 3% agarose gel. The electrophoresis pattern displayed different bands corresponding to the different genotypes. The CC genotype showed a single band at 214 bp, the TT genotype exhibited two bands at 171 bp and 43 bp, and the heterozygote genotype (CT) displayed three bands at 214 bp, 171 bp, and 43 bp.

Genotyping of rs737337 (T/C) was carried out via high-resolution melting curve (HRM) analysis on a RotorGene 6000 instrument. The HRM analysis was carried out with 5x HOT FIREPol EvaGreen HRM Mix (Solis BioDyne, Tartu, Estonia). Primer sequences that were used for the HRM analyses: F: GGGTGCACAGAGGACACG, R: TGGGTGGACGGTCACAAGGTCACAAG.

### Statistical analyses

We used SPSS 22.0 (IBM, New York, USA) for the statistical analysis. Mean and standard deviation (SD) were used to describe continuous Gaussian variables, while median (Q1–Q3) was used to describe continuous non-Gaussian variables. Allele frequency (%) was employed to compare NAFLD patients and healthy controls, and a chi-square test was used to determine if there were statistically significant differences in genotype distributions between the two groups. Multinomial logistic regression was used, with genotype as the factor and sex and age as variables, to estimate the likelihood of developing metabolic syndrome and its component disorders. The most prevalent genotypes served as a benchmark for comparison. Odds ratios (ORs) with 95% confidence intervals (CIs) were used to summarize the findings. All two-tailed tests were considered significant if the *P* value was less than 0.05.

## Results

### Comparing key demographic and biochemical characteristics of NAFLD patients and the control Group

In Table 1, we give the characteristics of the study participants, broken down according to whether they were part of the NAFLD group or the control group. The statistical analysis indicated that the proportion of men to women was significantly higher in both categories, and these two groups were not gender homogenous (*P* value < 0.05) (Table 1). However, the average age and BMI were found to be equal between the two groups, with no significant differences (*P* value < 0.05) (Table 2). Additionally, there was a significant difference in the RC between individuals with and without NAFLD. Specifically, RC is found to be higher in individuals diagnosed with fatty liver compared to those without the condition. Participants with NAFLD have significantly higher levels of FBG, TG, total cholesterol, insulin, HOMA-IR, and LDL compared to those without NAFLD. In contrast, the NAFLD group had a lower HDL cholesterol level than the control group. According to the data presented in Table 2, the LSM in the NAFLD groups was found to be ≥ 7, which indicates the F2 degree of fibrosis. This suggests that the NAFLD groups had moderate fibrosis, as determined by the LSM values. In contrast, the control group had LSM values below 7, indicating the absence of significant fibrosis. Therefore, the control group was categorized as having a lower degree of fibrosis compared to the NAFLD groups, based on the LSM criteria.

**Table 1:** The distribution of sex among patients with NAFLD and control patients. The number and percentage of male and female patients in each group are displayed

Sex	Group		T	*P* Value
	NAFLD	Control		
Male	134 (60/40%)	147 (73/10%)	7.72	0.005
Female	88 (39/60%)	54 (26/90%)		

**Table 2:** According to the research groups, the anthropometric and metabolic features of the individuals

Variable	Group				*P* Value
	NAFLD		Control		
	Mean ± SD	Median(Q1-Q3)	Mean ± SD	Median(Q1-Q3)	
Age	54.55 ± 7.82	53.00 (48.75–61.00)	55.56 ± 7.90	55.00 (49.00–62.00)	0.148
BMI	27.01 ± 4.24	26.98 (24.07–30.14)	26.39 ± 3.70	26.07 (23.76–28.78)	0.078
Systolic pressure (mmhg)	130.86 ± 19.16	128.00 (117.50–143.00)	127.31 ± 18.18	125.00 (115.00–137.00)	0.061
Diastolic pressure (mmhg)	80.00 ± 12.67	78.00 (72.50–88.00)	77.16 ± 12.73	75.00 (69.50–85.00)	0.002
FBG	100.25 ± 12.82	100.5 (90.75–110.00)	90.80 ± 11.19	125.00 (115.00–137.00)	< 0.001
Serum Insulin	8.69 ± 5.06	8.60 (5.00–11.33)	5.24 ± 3.91	75.00 (69.50–85.00)	< 0.001
HOMA IR	3.10 ± 2.17	2.83 (1.48–4.14)	1.24 ± 0.94	1.00 (0.54–1.59)	< 0.001
TG	151.77 ± 54.35	150.00 (117.50–143.00)	128.25 ± 51.37	119.00 (90.50–167.00)	< 0.001
Total Cholesterol	192.29 ± 45.19	197.5 (162.00–227.00)	165.18 ± 44.71	166.00 (136.50–194.00)	< 0.001
LDL Cholesterol	119.60 ± 35.89	118.00 (96.00–143.50)	100.30 ± 34.41	101.00 (77.00–123.50)	< 0.001
HDL Cholesterol	42.62 ± 8.00	42.00 (38.00–46.00)	44.65 ± 8.94	44.00 (39.00–50.00)	< 0.001
ALT	25.85 ± 11.61	23.80 (17.68–31.73)	21.02 ± 8.19	20.08 (14.25–26.70)	< 0.001
AST	23.41 ± 0.49	22.80 (18.90–27.80)	20.02 ± 0.46	18.80 (15.30–24.80)	< 0.001
RC	35.22 ± 17.12	35 (23–47)	27.82 ± 16.63	23 (16–38)	< 0.001
LSM	7.40 ± 2.57	7.2 (5.6–9.02)	5.49 ± 2.06	5 (4-6.45)	< 0.001

**Fig. 1:**
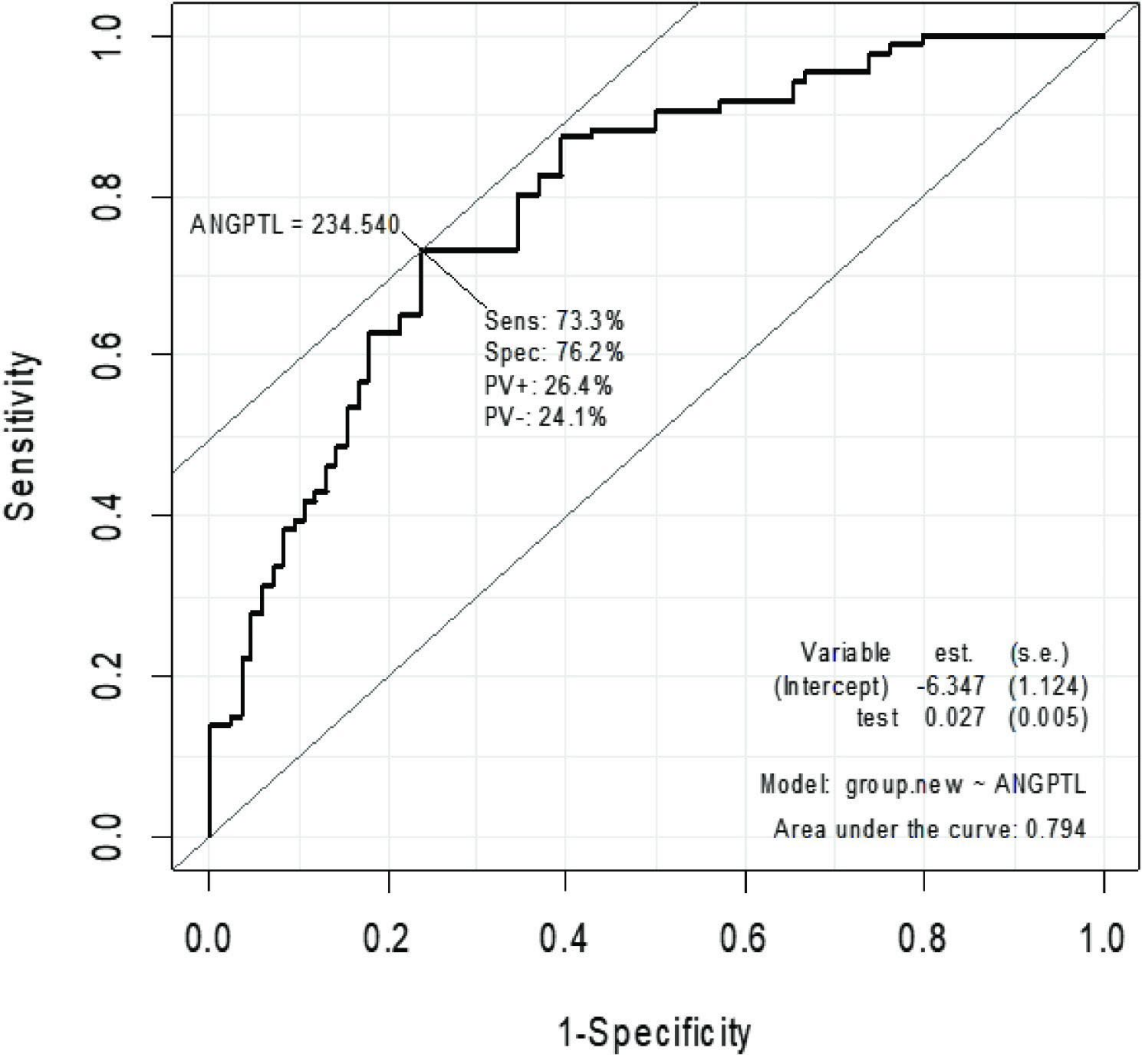
The figure displays the ROC curve depicting the correlation between NAFLD and ANGPTL8 protein. The curve shows the sensitivity of the ANGPTL8 protein in predicting the presence of NAFLD at different cutoff points. The area under the curve (AUC) is a measure of the accuracy of the test, with values ranging from 0.5 (no accuracy) to 1 (perfect accuracy)

The investigation of the serum level of ANGPTL8 revealed a significant increase in patients with NAFLD. The receiver operating characteristic (ROC) diagram demonstrated that introducing a cutoff point of 234.54 can distinguish between patients and healthy individuals, achieving a sensitivity of 73.30% and a specificity of 76.20%. The level below the graph is 80%, which is a significant difference of 0.5 (*P* value < 0.001) (Fig. 1).

### Uncovering the Genetic Landscape: analysis of SNP genotype and allele frequencies

Table 3 shows the genotype and allele frequency distribution of the ANGPTL8 gene SNPs, including the polymorphisms rs737337 (T > C) and rs2278426 (C > T).

**Table 3:** This table presents the distribution of genotype and allele frequencies for two SNPs, rs737337 and rs2278426, among the individuals who participated in the study

Polymorphism	Genotype	Group				*P* Value
		NAFLD		Control		
		Count	Percentage	Count	Percentage	
rs737337	TT	155	69.80	107	53.20	0.002
	TC	51	23.00	68	33.80	
	CC	16	7.20	26	12.90	
rs2278426	CC	164	73.90	167	83.10	0.045
	CT	52	23.40	28	13.90	
	TT	6	2.70	6	3.00	

When comparing NAFLD participants to control individuals, we observed a significantly higher T-allele frequency of the rs737337 polymorphism in the NAFLD group (p = 0.002). On the other hand, the C allele frequency of the rs2278426 polymorphism was slightly higher in the control group than in the NAFLD group (*P* = 0.045). These results suggest that the rs737337 and rs2278426 polymorphisms may be associated with NAFLD susceptibility (Table 4).

**Table 4:** The study participants’ genotypes and the allele frequency distribution of the SNPs rs737337 and rs2278426

SNP	Parameters	Allele	Groups		*P* Value	OR
			NAFLD	Control		
**Rs737337**	TT	0	67 (30.20)	94 (46.80)	< 0.001	2.032 (1.364–3.028)
		1	155 (69.80)	107 (53.20)		
	TC	0	171 (77.00)	133 (66.20)	0.013	0.583 (0.380–0.895)
		1	51 (23.00)	68 (33.80)		
	CC	0	206 (92.80)	175 (87.10)	0.049	0.523 (0.272–1.006)
		1	16 (7.20)	26 (12.90)		
**rs2278426**	CC	0	58 (26.10)	34 (16.90)	0.022	0.576 (0.358–0.926)
		1	164 (73.90)	167 (83.10)		
	CT	0	170 (76.60)	173 (86.10)	0.013	1.890 (1.140–3.134)
		1	52 (23.40)	28 (13.90)		
	TT	0	216 (97.30)	195 (97.00)	0.861	0.903 (0.286–2.846)
		1	6 (2.70)	6 (3.00)		

### Exploring the link between ANGPTL8 gene variants and anthropometric parameters in NAFLD patients

To investigate the differences in biochemical parameters based on variations in the ANGPTL8 gene, we conducted an analysis considering the genotypes of rs737337 (T > C) and rs2278426 (C > T). The results are presented in Table 5. After adjusting for age and sex, it was observed that participants with the TT genotype of the rs737337 SNP exhibited significantly higher levels of insulin (*P* < 0.014), HOMA-IR (p < 0.003), TG (*P* < 0.001), and ANGPTL8 (*P* < 0.001) compared to individuals with TC and CC genotypes. These findings suggest that the rs737337 TT genotype may be associated with increased levels of insulin, HOMA-IR, TG, and ANGPTL8 in this population. In addition to the observed differences in insulin, HOMA-IR, TG, and ANGPTL8 levels, it was found that individuals with the TT genotype of the rs737337 SNP had significantly higher levels of LDL cholesterol (*P* = 0.005) compared to those with the CC genotype. Furthermore, ALT levels were significantly higher in individuals with the TT genotype than in those with the TC genotype (*P* = 0.024). Although BMI, FBG, AST, and TC also showed a similar trend, these changes were not statistically significant. The HDL-cholesterol levels in individuals with the TT genotype were lower than those in individuals with the TC and CC genotypes, but this difference did not reach statistical significance. These findings indicate potential associations between the rs737337 SNP and LDL-cholesterol and ALT levels, suggesting a possible role of the ANGPTL8 gene variation in lipid metabolism and liver function. In contrast, the RC level was higher in the TT and TC genotypes than in the CC genotype (*P* > 0.05).

**Table 5:** The anthropometric and biochemical characteristics of the two research SNPs. Non-Gaussian variables are shown as medians with the first and third quartiles, whereas Gaussian variables are presented as the mean plus or minus SD. Statistical significance is indicated by a *P* value lower than 0.05. For rs737337 and rs2278426, the letters “a”, “b”, and “d” denote a substantial difference in comparison to the TT, CT, and CC genotypes, respectively

	Parameters	TT	TC	CC	*P* Value	TC + CC	*P* Value
**rs737337 (T/C)**	**N (M/F)**	262 (173/89)	119 (76/43)	42 (32/10)	0.339	161 (108/53)	0.824
	**Age (years)**	55 ± 8.12	58.23 ± 7.86	56.23 ± 7.97	0.355	57.43 ± 7.90	0.164
	**BMI (kg/m** ^**2**^ **)**	28.05 ± 3.88	25.89 ± 4.34	26.26 ± 4.41	0.343	26.03 ± 4.33	0.154
	**Insulin**	8.07 ± 5.11 d	5.37 ± 3.75	5.23 ± 3.94	0.039	5.31 ± 3.79	0.014
	**HOMA-IR**	2.67 ± 2.07 d	1.58 ± 1.34 a	1.58 ± 1.78	0.009	1.58 ± 1.51	0.003
	**AST**	22.85 ± 7.66	20.47 ± 6.32	21.91 ± 7.58	0.134	21.03 ± 6.82	0.048
	**ALT**	25.26 ± 12.06	19.51 ± 6.97 a	23.8 ± 8.53	0.024	21.20 ± 7.84	0.009
	**ANGPTL8**	252.39 ± 50.02	223.95 ± 45.54 a	199.22 ± 41.96 a	˂0.001	214.21 ± 45.49	0.000
	**SBP (mmHg)**	133.27 ± 19.27	130.35 ± 19.85	124.38 ± 16	0.373	128.0 ± 18.53	0.314
	**DBP (mmHg)**	79.6 ± 12.04 d	81.85 ± 13.43	73.35 ± 11.15	0.037	78.50 ± 13.17	0.241
	**Glucose**	98.73 ± 13.65	94.65 ± 12.3	94.31 ± 13.04	0.147	94.51 ± 12.5	0.056
	**Triglyceride**	151.8 ± 53.53 d	141.73 ± 45 d	106.96 ± 60.38	˂0.001	128.03 ± 53.94	0.000
	**Total Cholesterol**	184.64 ± 44.75	174.58 ± 53.36 d	138.77 ± 49.5 a	˂0.001	160.46 ± 54.41	0.001
	**HDL Cholesterol**	43.34 ± 7.35	42.43 ± 7.44	45.92 ± 10.94	0.115	43.80 ± 9.06	0.228
	**LDL Cholesterol**	118.09 ± 34.76 d	107.63 ± 40.21 d	80.5 ± 35.28	˂0.001	96.93 ± 40.33	0.005
	**RC**	32 ± 17.39	32.16 ± 17.04	25.74 ± 16.36	0.088	30.57 ± 17.04	0.27
	**LSM**	6.56 ± 2.49	6.33 ± 2.47	6.50 ± 2.92	0.628	6.37 ± 2.59	0.345
**rs2278426(C/T)**	**Parameters**	**CC**	**CT**	**TT**	***P*** **Value**	**CT + TT**	***P*** **Value**
	**N (M/F)**	12 (7/5)	80 (56/24)	331 (218/113)	0.651	92 (63/29)	0.638
	**Age (years)**	54.33 ± 9.18	56.27 ± 8.78	55.95 ± 7.93	0.420	55.97 ± 8.75	0.783
	**BMI (kg/m** ^**2**^ **)**	27.87 ± 5.53	28.04 ± 3.9	27.04 ± 4.18	0.964	28.02 ± 4.11	0.994
	**Insulin**	6.42 ± 6.41	8.46 ± 6.08	6.65 ± 4.34	0.474	8.15 ± 6.09	0.363
	**HOMA-IR**	1.7 ± 1.55	2.96 ± 2.47	2.08 ± 1.77	0.285	2.76 ± 2.38	0.173
	**AST**	28.7 ± 13.34	22.41 ± 7.27	21.77 ± 6.98	0.597	23.38 ± 8.55	0.893
	**ALT**	33.8 ± 19.88	23.11 ± 10.64	23.35 ± 10.14	0.891	24.75 ± 12.75	0.698
	**ANGPTL8**	325.18 ± 64.77 d	260.58 ± 48.37 d, a	227.56 ± 46.25 d, a	˂0.001	270.52 ± 55.5	0.000
	**SBP (mmHg)**	131.17 ± 20.79	137.03 ± 15.61	129.74 ± 19.68	0.388	136.13 ± 16.33	0.230
	**DBP (mmHg)**	80.67 ± 11.24	82.06 ± 10.56	78.37 ± 12.92	0.775	81.85 ± 10.52	0.476
	**Glucose**	90.5 ± 3.73	99.03 ± 12.92	96.89 ± 13.66	0.528	97.72 ± 12.34	0.656
	**Triglyceride**	146.5 ± 38.54	154.33 ± 58.55	139.33 ± 54.34	0.171	153.12 ± 55.592	0.061
	**Total Cholesterol**	210 ± 48.76	185.0606 ± 54.33	171.09 ± 48.32	0.363	188.89 ± 53.68	0.547
	**HDL Cholesterol**	41.83 ± 9.33	44.8485 ± 6.58	43.26 ± 8.33	0.853	44.38 ± 7.00	0.825
	**LDL Cholesterol**	107.6 ± 37.4	114.57 ± 41.39	132.00 ± 37.59	0.837	103.56 ± 36.91	0.073
	**RC**	30.81 ± 17.23^b^	34.98 ± 16.92	27.27 ± 17.92	0.049	35.55 ± 17.06	0.017
	**LSM**	6.38 ± 2.49	6.95 ± 2.68	6.43 ± 2.76	0.257	6.88 ± 2.68	0.143

**a: TT, b: CT, d: CC**

With regard to the rs2278426 SNP, the levels of ANGPTL8 and RC significantly increased in CC and CT in comparison to TT. On the other hand, the levels of insulin, HOMA-IR, FBG, and HDL cholesterol in individuals with the CC genotype were found to be higher than those in individuals with the TT genotype, although the difference was not statistically significant (*P* > 0.05).

The analysis of the association between LSM and NAFLD did not yield significant correlations for either SNP (rs2278426 and rs737337) in this study. Despite investigating the potential relationship between these genetic variants and NAFLD-related parameters, our findings did not reveal a statistically significant correlation between LSM values and the ANGPTL8 gene SNPs (Table 5).

### Association of ANGPTL8 gene variants with NAFLD components

Participants were stratified into distinct categories based on predetermined cutoff values for various physiological parameters, including obesity (BMI > 30), elevated levels of AST (> 35 IU/L), elevated levels of ALT (> 38 IU/L), hypertension (systolic blood pressure/diastolic blood pressure > 130/85), low levels of HDL cholesterol < 40 mg/dL for men and < 50 mg/dL for women), hypertriglyceridemia (> 150 mg/dL), hypercholesterolemia (> 200 mg/dL), high glucose levels (> 126 mg/dL), and NAFLD. Subsequently, the impact of various variants of the ANGPTL8 gene on the categories mentioned above was analyzed using logistic regression analyses of the genotypes/alleles in the SNP rs737337 and SNP rs2278426, which represent the risk of developing different components of NAFLD. The results of these analyses are presented in Table 5.

As demonstrated in Table 6, the combined TC + CC genotype and C allele of the rs737337 polymorphism were significantly associated with a decreased risk of obesity. This association was observed in both the univariate model (OR 0.88, 95% confidence interval [CI]: 0.55–1.43, *P* = 0.625) and after adjusting for age and sex (OR 0.903, 95% CI: 0.558–1.462, p = 0.679). These results suggest that individuals with the TC + CC genotype and carrying the C allele may have a lower likelihood of developing obesity compared to those with the TT genotype. However, it is important to note that the associations were not significant. Similar trends were observed for ALT and AST parameters. In the univariate model, the TC + CC genotype and C allele of rs737337 were associated with a decreased risk of elevated ALT levels (OR 0.532, 95% CI: 0.252–1.123, *P* = 0.098), and after adjustment for age and gender, the association remained (OR 0.555, 95% CI: 0.257–1.198, *P* = 0.134). Similarly, for AST levels, the TC + CC genotype and C allele showed a decreased risk, although not statistically significant (OR 0.581, 95% CI: 0.182–1.857, *P* = 0.36 in the univariate model; OR 0.583, 95% CI: 0.177–1.921, *P* = 0.375 after adjustment). These findings suggest a potential protective effect of the TC + CC genotype and C allele against elevated ALT and AST levels, indicating a possible role of the rs737337 polymorphism in liver function. However, larger studies are required to confirm these associations and better understand the underlying mechanisms.

**Table 6:** The odds ratio (95% confidence interval) indicating the risk of NAFLD score associated with gene variants in the rs737337 SNP. The univariate values are represented by OR^a^ and p^a^, while the values after adjustment for age and gender are represented by OR^b^ and *P*^b^. To determine the presence of obesity, a BMI greater than 30 kg/m^2^ was considered; hypertension was indicated by blood pressure equal to or greater than 130/85 mmHg; elevated levels of AST (> 35 IU/L); elevated levels of ALT (> 38 IU/L); low HDL-C was defined as HDL-cholesterol levels lower than 40 mg/dL for men and lower than 50 mg/dL for women; hypertriglyceridemia was identified if triglyceride levels were equal to or greater than 150 mg/dL; hypercholesterolemia was defined as total cholesterol greater than 200 mg/dL; and high glucose was considered present if fasting glucose levels were equal to or greater than 126 mg/dL. The presence of NAFLD was determined based on the IDF criteria. A *P* value less than 0.05 was considered statistically significant

Parameters		Yes	β	OR (95% CI)^a^	*P* ^a^	OR (95% CI)^b^	*P* ^b^
Obes (kg/m^2^)	TT	59	1				
	TC	24	0.14	1.15 (0.67–1.96)	0.607	1.13 (0.66–1.93)	0.648
	CC	9	0.064	1.06 (0.48–2.35)	0.875	1.04 (0.47–2.31)	0.918
	TC + CC	33	-0.12	0.88 (0.55–1.43)	0.625	0.90 (0.56–1.46)	0.676
	T						
	C	33	-0.12	0.887 (0.549–1.434)	0.625	0.903 (0.558–1.462)	0.679
High AST	TT	11	1				
	CC	4	-0.231	0.794 (0.247–2.546)	0.698	0.744 (0.224–2.472)	0.629
	TC	0	-18.07	0.000	0.998	0.000	0.997
	TC + CC	4	-0.542	0.581 (0.182–1.857)	0.36	0.583 (0.177–1.921)	0.375
	T						
	C	4	-0.542	0.581 (0.182–1.857)	0.36	0.583 (0.177–1.921)	0.375
High ALT	TT	29	1				
	CC	7	-0.689	0.502 (0.213–1.181)	0.115	0.493 (0.204–1.189)	0.115
	TC	3	-0.481	0.618 (0.180–2.127)	0.445	0.777 (0.218–2.776)	0.698
	TC + CC	10	-0.631	0.532 (0.252–1.123)	0.098	0.555 (0.257–1.198)	0.134
	T						
	C	10	-0.631	0.532 (0.252–1.123)	0.098	0.555 (0.257–1.198)	0.134
SBP (mmHg)	TT	119	1				
	CC	54	-0.002	0.998 (0.646–1.543)	0.994	0.982 (0.634–1.522)	0.935
	TC	15	-0.404	0.668 (0.339–1.313)	0.242	0.687 (0.348–1.357)	0.279
	TC + CC	69	-0.104	0.901 (0.607–1.339)	0.607	0.897 (0.602–1.336)	0.593
	T						
	C	69	-0.104	0.901 (0.607–1.339)	0.607	0.897 (0.602–1.336)	0.593
DBP (mmHg)	TT	87	1				
	CC	41	0.056	1.057 (0.669–1.670)	0.811	1.059 (0.669–1.676)	0.808
	TC	10	-0.464	0.629 (0.295–1.338)	0.228	0.654 (0.306–1.397)	0.273
	TC + CC	51	-0.07	0.933 (0.613–1.419)	0.745	0.943 (0.619–1.439)	0.787
	T						
	C	51	-0.070	0.933 (0.613–1.419)	0.745	0.943 (0.619–1.439)	0.787
High Glucose	TT	1	1				
	CC	0	-15.638	0.000	0.997	0.000	0.996
	TC	0	-15.638	0.000	0.998	0.000	0.998
	TC + CC	0	-15.638	0.000	0.996	0.000	0.995
	T						
	C	0	-15.638	0.000	0.996	0.000	0.995
Hyper Triglyceridemia	TT	130	1				
	CC	45	-0.482	0.617 (0.397–0.961)	0.033	0.629 (0.403–0.981)	0.041
	TC	7	-1.594	0.203 (0.087–0.474)	0.000	0.203 (0.087–0.476)	0.000
	TC + CC	52	-0.725	0.484 (0.322–0.730)	0.001	0.492 (0.326–0.742)	0.001
	T						
	C	52	-0.725	0.484 (0.322–0.73)	0.001	0.492 (0.326–0.742)	0.001
Low HDL-C	TT	137	1				
	CC	62	-0.008	0.992 (0.643–1.531)	0.973	0.883 (0.531–1.467)	0.630
	TC	15	-0.679	0.507 (0.258–0.997)	0.049	0.514 (0.233–1.134)	0.099
	TC + CC	77	-0.179	0.836 (0.565–1.239)	0.373	0.768 (0.484–1.219)	0.263
	T						
	C	77	-0.179	0.836 (0.565–1.239)	0.373	0.768 (0.484–1.219)	0.263
High LDL	TT	84	1				
	CC	40	0.07	1.073 (0.677-1.70)	0.764	1.077 (0.679–1.708)	0.753
	TC	4	-1.50	0.223 (0.077–0.645)	0.006	0.221 (0.076–0.641)	0.005
	TC + CC	44	-0.227	0.797 (0.517–1.229)	0.304	0.798 (0.517–1.231)	0.308
	T						
	C	44	-0.227	0.797 (0.517–1.229)	0.304	0.798 (0.517–1.231)	0.038
HOMA-IR	TT	98	1				
	CC	9	-0.784	0.456 (0.21–0.994)	0.048	0.468 (0.215–1.02)	0.057
	TC	36	-0.32	0.726 (0.45–1.15)	0.176	0.724 (0.45–1.15)	0.175
	TC + CC	45	-0.432	0.649 (0.42–0.99)	0.047	0.653 (0.42-1)	0.05
	T						
	C	45	-0.432	0.649 (0.42–0.99)	0.047	0.653 (0.42-1)	0.05
RC	TT	204	1				
	CC	15	-0.67	0.51(0.255–1.03)	0.059	0.54(0.26–1.09)	0.085
	TC	30	-0.17	0.84(0.51–1.40)	0.51	0.84 (0.51–1.41)	0.526
	TC + CC	116	-0.31	0.73(0.46–1.15)	0.733	0.74(0.47–1.17)	0.746
	T						
	C	116	-0.31	0.73(0.46–1.15)	0.733	0.74(0.47–1.17)	0.746
NAFLD	TT	155	1				
	CC	51	-0.658	0.518 (0.334–0.803)	0.003	0.510 (0.327–0.796)	0.003
	TC	16	-0.856	0.425 (0.217–0.83)	0.012	0.451 (0.230–0.888)	0.021
	TC + CC	67	-0.709	0.492 (0.330–0.733)	0.000	0.494 (0.330–0.740)	0.001
	T						
	C	67	-0.709	0.492 (0.330–0.733)	0.000	0.494 (0.330–0.740)	0.001

As shown in Table 7, the findings of this study suggest that the TC + CC genotype and C allele of rs2278426 are significantly associated with an increased risk of obesity, both before and after controlling for age and gender. In the univariate model, the ORs for these associations were 0.88 (95% CI: 0.40–0.93; *P* = 0.02) and 0.58 (95% CI: 0.39–0.86; *P* = 0.007), respectively. After adjusting for age and gender, the ORs remained significant at 0.65 (95% CI: 0.42–0.99; *P* < 0.05) and 0.62 (95% CI: 0.41–0.93; *P* = 0.02), respectively. Interestingly, the results for ALT and AST showed a similar trend, with ORs not reaching statistical significance in either the univariate or adjusted models. Specifically, the ORs for ALT in the univariate and adjusted models were 1.088 (95% CI: 0.497–2.382; *P* = 0.833) and 1.189 (95% CI: 0.531–2.666; *P* = 0.674), respectively. The corresponding ORs for AST were 1.322 (95% CI: 0.411–4.254; *P* = 0.639) and 1.451 (95% CI: 0.431–1.886; *P* = 0.548), respectively.

**Table 7:** The odds ratio (95% confidence interval) indicating the risk of NAFLD score associated with gene variants in the rs2278426 SNP. The univariate values are represented by OR^a^ and p^a^, while the values after adjustment for age and gender are represented by OR^b^ and p^b^. To determine the presence of obesity, a BMI greater than 30 kg/m^2^ was considered; hypertension was indicated by blood pressure equal to or greater than 130/85 mmHg; elevated levels of AST (> 35 IU/L); elevated levels of ALT (> 38 IU/L); low HDL-C was defined as HDL-cholesterol levels lower than 40 mg/dL for men and lower than 50 mg/dL for women; hypertriglyceridemia was identified if triglyceride levels were equal to or greater than 150 mg/dL; hypercholesterolemia was defined as total cholesterol greater than 200 mg/dL; and high glucose was considered present if fasting glucose levels were equal to or greater than 126 mg/dL. The presence of NAFLD was determined based on the IDF criteria. A *P* value less than 0.05 was considered statistically significant

Parameters		Yes	β	OR (95% CI)^a^	*P* ^a^	OR (95% CI)^b^	*P* ^b^
Obes (kg/m2)	CC	73	1				
	CT	16	-0.124	0.884 (0.48–1.62)	0.68	0.893 (0.48–1.64)	0.716
	TT	3	0.164	1.178 (0.31–4.46)	0.809	1.13 (0.29–4.30)	0.857
	CT + TT	19	-0.084	0.92 (0.521–1.623)	0.773	0.924 (0.523–1.632)	0.785
	C						
	T	19	-0.084	0.92 (0.521–1.623)	0.773	0.924 (0.523–1.632)	0.785
High AST	CC	11	1				
	CT	2	− 0.0293	0.746 (0.162–3.434)	0.707	0.814 (0.17–3.907)	0.797
	TT	2	1.761	5.81 (1.137–29.779)	0.035	6.329 (1.003–39.950)	0.050
	CT + TT	4	0.279	1.322 (0.411–4.254)	0.639	1.451 (0.431–4.886)	0.548
	C						
	T	4	0.279	1.322 (0.411–4.254)	0.639	1.451 (0.431–4.886)	0.548
High ALT	CC	30	1				
	CT	6	-0.206	0.814 (0.327–2.026)	0.658	0.924 (0.363–2.356)	0.869
	TT	3	1.207	3.34 (0.859–13.024)	0.082	2.857 (0.692–11.801)	0.147
	CT + TT	9	0.084	1.088 (0.497–2.382)	0.833	1.189 (0.531–2.666)	0.674
	C						
	T	9	0.084	1.088 (0.497–2.382)	0.833	1.189 (0.531–2.666)	0.674
SBP (mmHg)	CC	146	1				
	CT	38	0.137	1.146 (0.703–1.870)	0.584	1.164 (0.711–1.906)	0.545
	TT	4	-0.456	0.634 (0.187–2.145)	0.463	0.620 (0.182–2.119)	0.446
	CT + TT	42	0.062	1.064 (0.669–1.693)	0.792	1.075 (0.674–1.715)	0.760
	C						
	T	42	0.062	1.064 (0.669–1.693)	0.792	1.075 (0.674–1.715)	0.76
DBP (mmHg)	CC	101	1				
	CT	32	0.417	1.518 (0.916–2.515)	0.105	1.553 (0.935–2.579)	0.089
	TT	5	0.486	1.627 (0.504–5.247)	0.416	1.557 (0.480–5.054)	0.461
	CT + TT	37	0.427	1.532 (0.950–2.471	0.080	1.553 (0.961–2.510)	0.072
	C						
	T	37	0.427	1.532 (0.95–2.471)	0.080	1.553 (0.961–2.510)	0.072
High Glucose	CC	1	1				
	CT	0	-15.404	0.000	0.997	0.000	0.997
	TT	0	-15.404	0.000	0.999	0.000	0.999
	CT + TT	0	-15.404	0.000	0.997	0.000	0.997
	C						
	T	0	-15.404	0.000	0.997	0.000	0.997
Hyper Triglyceridemia	CC	138	1				
	CT	37	0.185	1.203 (0.737–1.966)	0.460	1.217 (0.743–1.993)	0.436
	TT	7	0.672	1.958 (0.609–6.298)	0.260	1.883 (0.582–6.090)	0.291
	CT + TT	44	0.248	1.282 (0.806–2.038)	0.294	1.288 (0.808–2.052)	0.287
	C						
	T	44	0.248	1.282 (0.806–2.038)	0.294	1.288 (0.808–2.052)	0.287
Low HDL-C	CC	170	1				
	CT	37	-0.205	0.815 (0.499–1.330)	0.412	0.834 (0.470–1.479)	0.534
	TT	7	0.282	1.326 (0.412–4.262)	0.636	1.304 (0.328–5.186)	1.304
	CT + TT	44	-0.141	0.868 (0.547–1.378)	0.549	0.882 (0.513–1.517)	0.649
	C						
	T	44	-0.141	0.868 (0.547–1.378)	0.549	0.882 (0.513–1.517)	0.649
High LDL	CC	100	1				
	CT	25	0.049	1.050 (0.619–1.780)	0.856	1.050 (0.619–1.781)	0.856
	TT	3	-0.261	0.770 (0.204–2.904)	0.700	0.767 (0.203–2.898)	0.696
	CT + TT	28	0.011	1.011 (0.612–1.670)	0.967	1.010 (0.611–1.669)	0.969
	C						
	T	28	0.011	1.011 (0.612–1.670)	0.967	1.01 (0.971–1.024)	0.827
HOMA-IR	CC	109					
	CT	31	0.253	1.289 (0.77–2.13)	0.325	1.308 (0.87–2.17)	0.298
	TT	3	-0.387	0.679 (0.18–2.55)	0.567	0.655 (0.17–2.47)	0.533
	CT + TT	34	0.17	1.194 (0.73–1.93)	0.471	1.204 (0.74–1.95)	0.451
	C						
	T	34	0.17	1.194 (0.73–1.93)	0.471	1.204 (0.74–1.95)	0.451
RC	CC	251					
	CT	58	-0.17	0.84(0.48–1.45)	0.53	0.86(0.49–1.49)	0.86
	TT	11	1.125	3.51(0.44–27.57)	0.23	3.30(0.41–26.11)	0.25
	CT + TT	69	-0.04	0.95(0.56–1.63)	0.87	0.96(0.56–1.66)	0.909
	C						
	T	69	-0.04	0.95(0.56–1.63)	0.87	0.96(0.56–1.66)	0.909
NAFLD	CC	164	1				
	CT	52	0.637	1.891 (1.139–3.141)	0.014	1.988 (1.189–3.325)	0.009
	TT	6	0.018	1.018 (0.322–3.222)	0.975	0.932 (0.290–3.003)	0.907
	CT + TT	58	0.522	1.737 (1.080–2.793	0.023	1.793 (1.108-2.900)	0.17
	C						
	T	58	0.522	1.737 (1.080–2.793	0.023	1.793 (1.108-2.900)	0.17

## Discussion

NAFLD has become a significant global health problem, emerging as a leading cause of chronic liver diseases. In response to this growing concern, scientists have directed their efforts toward preventive and therapeutic approaches to tackle NAFLD. Among these strategies, the involvement of ANGPTL8, a protein implicated in the development of NAFLD, has gained attention. However, research on the association between ANGPTL8 and NAFLD is limited, particularly in Iran.

In this study, our main objective was to investigate the potential role of two ANGPTL8 gene polymorphisms, specifically rs2278426 (C/T) and rs737337 (T/C), in the susceptibility to NAFLD among Iranian adults. In this study group, our findings revealed a notable association between the rs2278426 variant and an elevated risk of NAFLD. However, we did not observe a significant association between the rs737337 variant and NAFLD risk. These results suggest that genetic variations in the ANGPTL8 gene may contribute to the development of NAFLD in Iranian adults. The identified association with the rs2278426 variant implies that this specific genetic variant might influence the susceptibility to NAFLD in this population. This finding highlights the potential relevance of ANGPTL8 as a target for preventive and therapeutic approaches in managing NAFLD among Iranian adults.

Our study investigated the potential associations between ANGPTL8 gene variants (rs737337 and rs2278426) and certain metabolic parameters, such as hypercholesterolemia, hyperglycemia, ALT, and AST levels. We observed interesting trends in the data, but these associations were not statistically significant after adjusting for age and sex. Specifically, individuals with the C allele of the rs737337 variant tended to have a lower likelihood of developing hypercholesterolemia and hyperglycemia, suggesting a potential protective effect. Similarly, individuals with the T allele of the rs2278426 variant displayed a tendency toward a lower likelihood of developing hyperglycemia, indicating a potential protective role. Interestingly, we observed a trend toward higher ALT and AST levels and hypercholesterolemia in individuals with the T allele of the rs2278426 variant. However, these associations were not statistically significant, even after accounting for age and sex. Although these findings did not reach statistical significance, they provide valuable insights into the potential relationships between ANGPTL8 gene variants and metabolic parameters in the context of NAFLD. Further research with larger sample sizes may be needed to validate and better understand these associations in the Iranian population.

Our investigation revealed that ANGPTL8 gene variations were substantially linked with total cholesterol and TG levels. ANGPTL8 is known to play a vital role in lipid metabolism. Additionally, the T allele of SNPs rs2278426 (C/T) enhanced the likelihood of having greater levels of ALT and AST. The serum levels of ALT and AST have been regarded as reliable and sensitive markers of liver disease. ANGPTL8 regulates lipid accumulation in the liver and promotes autophagy. As a result of lipid accumulation in the liver, ALT and AST blood levels rise, which is one of the most predictive indicators of NAFLD and parenchymal damage. A recent study showed that increased ANGPTL8 expression can raise serum TG levels, while recombinant ANGPTL8 inhibits LPL activity [22]. ANGPTL8 inhibition of LPL in cardiac and skeletal muscle is also linked to ANGPTL8.

The coexpression of ANGPTL8 and ANGPTL3 has been shown to have an additional impact on serum TG levels. Moreover, various ethnic groups have reported differences in the frequencies of ANGPTL8 gene variants and their relationship with blood lipid levels. While certain studies have identified a negative correlation between ANGPTL8 and TG in patients with T2DM, conflicting findings have also been reported [23, 24]. Several coding variations in ANGPTL3 have been investigated and found to be associated with reduced plasma TG levels, independent of other metabolic features. These variations primarily result in a loss of function, showing that the deactivation of ANGPTL3 lowers TG levels. Numerous SNPs close to the ANGPTL3 gene (rs1748195, rs12130333, and rs2131925) have been linked to plasma TG levels, according to significant genome-wide association studies (GWASs) that looked into common genetic variants associated with lipid traits in large populations [25–27].

Further research is needed to determine the role of ANGPTL8 in the development of NAFLD. ANGPTL8 has potential as a target for β-cell regenerative therapy, as it can induce β-cell proliferation [28]. Previous studies suggest that ANGPTL8 is upregulated in T2DM individuals, possibly due to increased insulin production through β-cell proliferation [29]. While some reports suggest that insulin induces ANGPTL8 expression, there is evidence to suggest that ANGPTL8 is not associated with β-cell proliferation. Mice lacking ANGPTL8 demonstrated typical glucose metabolism in the presence of insulin resistance [8].

Alenad and colleagues conducted a study in Saudi adults to examine the association between gene variants in SNPs rs737337 (T/C) and rs2278426 (C/T) and the risk of metabolic syndrome (MetS) and its components. Interestingly, they found that these variants are linked to a reduced risk of hypercholesterolemia and hyperglycemia, which are components of MetS. These results support the growing body of evidence suggesting that ANGPTL8 plays a crucial role in lipid and glucose metabolism. The dysregulation of these processes is a contributing factor in the development of MetS [11].

Our research revealed a relationship between a decreased risk of hyperglycemia and the C-allele and T-allele of SNPs rs737337 (T/C) and rs2278426 (C/T). Although the specific method by which sequence variation alters the function of ANGPTL8 is still unknown, it is plausible that changes in the charge of the amino acid residue from arginine to tryptophan might affect the protein structure. While research suggests that the role of ANGPTL8 in glucose metabolism extends beyond β-cell proliferation, further study is necessary to confirm this. In our study, older participants exhibited typical characteristics of NAFLD, including central obesity and elevated blood pressure levels, glucose, ALT, AST, TG, and HOMA-IR, with decreased HDL cholesterol levels. The C-allele variant of SNP rs737337 (T/C) was associated with a lower risk of NAFLD, consistent with studies demonstrating increased ANGPTL8 expression in T2DM and/or obese individuals. Additionally, there is mounting evidence of a positive association between circulating ANGPTL8 levels, insulin resistance, and T2DM [30].

While the associations between the TC + CC genotype and C allele of rs2278426 with ALT and AST levels did not reach statistical significance in this study, some trends suggest that further investigation is warranted. Specifically, the ORs for ALT and AST showed a similar trend as those for obesity, albeit not statistically significant. These trends indicate that there may be a potential relationship between these genetic factors and ALT or AST levels. Nevertheless, it is important to acknowledge that the limited sample size of this study may have contributed to the lack of statistical significance. Therefore, it is crucial to conduct additional research with larger sample sizes to validate these findings and better understand the underlying mechanisms.

RC has recently been considered a promising biomarker for assessing cardiovascular risk [31]. Additionally, cardiovascular disease is a leading cause of mortality in NAFLD patients [32]. Huang et al. showed a positive association between RC and long-term mortality in individuals with metabolic-associated fatty liver disease (MAFLD) [33]. RC is determined by the cholesterol content in VLDL and chylomicron remnants [34], with chylomicron remnants being more prevalent after a meal, while VLDL remnants exist in both postprandial and fasting states [34]. Our study focused on fasting individuals, indicating that the measured RC primarily reflects the cholesterol content in VLDL remnants. Our findings support the notion that increased VLDL production and secretion in NAFLD patients contribute to elevated RC levels, reinforcing previous research. This study represents the first report demonstrating an association between ANGPTL8 SNPs and RC.

Considering the interplay between ANGPTL8 and ANGPTL4, it is plausible that ANGPTL4 may also have implications in the development and progression of NAFLD [9]. Since NAFLD is closely linked to dysregulated lipid metabolism and insulin resistance, ANGPTL4’s involvement in these processes may contribute to the pathogenesis of NAFLD. Further research is needed to elucidate the exact mechanisms and implications of ANGPTL4 in the pathogenesis of NAFLD and its interaction with ANGPTL8.

### Study strengths and limitations

This study’s findings present intriguing insights into the association between specific gene variants (rs737337 and rs2278426) and NAFLD within a distinct Iranian adult population. However, it is crucial to recognize the limitations inherent in this research. The study’s focus on a single ethnicity highlights the need for caution when extending these findings to broader ethnic groups. Additionally, the absence of data on circulating betatrophin and insulin levels underscores the necessity for further investigation into their role in NAFLD among Iranian adults. The study’s call for a separate exploration of these factors and the challenge of estimating SNP allele prevalence underscore the importance of refining future research. Despite these limitations, this study lays a foundation for deeper insights into gene variant associations with NAFLD and points the way for future inquiries.

## Conclusion

In conclusion, the ANGPTL8 gene SNPs rs2278426 and rs737337 are associated with NAFLD-related parameters, including ALT and AST levels, hyperTG, and lipid profiles. However, rs2278426 demonstrated a higher association with NAFLD than rs737337. This suggests that ANGPTL8 may play a role in the pathogenesis of NAFLD. Further studies are needed to elucidate the underlying mechanisms and confirm the clinical relevance of these findings. These SNPs could potentially serve as biomarkers for the early diagnosis, prognosis, and personalized treatment of NAFLD. Overall, these results contribute to a better understanding of the genetic basis of NAFLD and highlight the importance of personalized medicine in managing this complex disease.

### Electronic supplementary material

Below is the link to the electronic supplementary material.

Supplementary Material 1

## Data Availability

Not applicable.

## Declarations

(https://ethics.research.ac.ir/ProposalCertificateEn.php?id=216263&Print=true&NoPrintHeader=true&NoPrintFooter=true&NoPrintPageBorder=true&LetterPrint=true)
