# Supplementary material for: Genetic predisposition to nonalcoholic fatty liver disease: insights from ANGPTL8 gene variants in Iranian adults
Source: Lipids Health Dis. 2023 Sep 7;22:147. doi: 10.1186/s12944-023-01905-0 (PMC10483745; doi:10.1186/s12944-023-01905-0)
Supplement: Supplementary file 1 — Supplementary Material 1 [file 12944_2023_1905_MOESM1_ESM.docx]

**Abbreviations List:**

**NAFLD**: Non-alcoholic fatty liver disease

**NASH**: Nonalcoholic Steatohepatitis

**T2DM**: Type 2 diabetes mellitus

**ANGPTL8**: Angiopoietin-like protein 8

**TG**: Triacylglycerol

**MAF**: Minor allele frequency

**SNP**: Single nucleotide polymorphism

**BMI**: Body mass index

**HOMA-IR**: Homeostasis model assessment of insulin resistance

**HDL**: High-density lipoprotein

**LDL**: Low-density lipoprotein

**VLDL**: Very low-density lipoprotein

**AST:** Aspartate transaminase

**ALT:** Alanine transaminase

**SD**: Standard deviation

**FBG:** Fasting blood glucose

**RC:** Remnant Cholesterol

**LSM**: Liver stiffness measurement
